# Proteins of novel lactic acid bacteria from *Apis mellifera mellifera*: an insight into the production of known extra-cellular proteins during microbial stress

**DOI:** 10.1186/1471-2180-13-235

**Published:** 2013-10-22

**Authors:** Èile Butler, Magnus Alsterfjord, Tobias C Olofsson, Christofer Karlsson, Johan Malmström, Alejandra Vásquez

**Affiliations:** 1 Medical Microbiology, Department of Laboratory Medicine, Lund University, Sölvegatan 23, Lund SE-223 62, Sweden; 2 Department of Immunotechnology, Lund University BMC D13, Lund SE-221 84, Sweden; 3 Division of Infection Medicine, Department of Clinical Sciences, Lund University, BMC, B14, Lund 221 84, Sweden

**Keywords:** Lactic acid bacteria, Symbionts, Microbial stress, Proteomics, Honeybee

## Abstract

**Background:**

Lactic acid bacteria (LAB) has been considered a beneficial bacterial group, found as part of the microbiota of diverse hosts, including humans and various animals. However, the mechanisms of how hosts and LAB interact are still poorly understood. Previous work demonstrates that 13 species of *Lactobacillus* and *Bifidobacterium* from the honey crop in bees function symbiotically with the honeybee. They protect each other, their hosts, and the surrounding environment against severe bee pathogens, bacteria, and yeasts. Therefore, we hypothesized that these LAB under stress, i.e. in their natural niche in the honey crop, are likely to produce bioactive substances with antimicrobial activity.

**Results:**

The genomic analysis of the LAB demonstrated varying genome sizes ranging from 1.5 to 2.2 mega-base pairs (Mbps) which points out a clear difference within the protein gene content, as well as specialized functions in the honeybee microbiota and their adaptation to their host. We demonstrate a clear variation between the secreted proteins of the symbiotic LAB when subjected to microbial stressors. We have identified that 10 of the 13 LAB produced extra-cellular proteins of known or unknown function in which some are arranged in interesting putative operons that may be involved in antimicrobial action, host interaction, or biofilm formation. The most common known extra-cellular proteins secreted were enzymes, DNA chaperones, S-layer proteins, bacteriocins, and lysozymes. A new bacteriocin may have been identified in one of the LAB symbionts while many proteins with unknown functions were produced which must be investigated further.

**Conclusions:**

The 13 LAB symbionts likely play different roles in their natural environment defending their niche and their host and participating in the honeybee’s food production. These roles are partly played through producing extracellular proteins on exposure to microbial stressors widely found in natural occurring flowers. Many of these secreted proteins may have a putative antimicrobial function. In the future, understanding these processes in this complicated environment may lead to novel applications of honey crop LAB proteins.

## Background

Lactic acid bacteria (LAB), generally considered beneficial microorganisms, are found in diverse environments as part of human, animal, insect, and plant microbiomes and as microorganisms used in food applications. LAB are described as a biologically defined group rather than a taxonomically separate group [1,2]. The majority are non-pathogenic gram-positive bacteria that produce lactic acid during carbohydrate hexose sugar metabolism. However, there are known pathogenic species, most of which are found in the genus *Streptococcus*[3]. LAB include *Lactobacillus*, *Bifidobacterium*, *Lactococcus*, *Aerococcus*, *Leuconostoc*, *Oenococcus*, and *Pediococcus* that are functionally quite diverse [1,3].

*Bifidobacterium* are classified as LAB biologically rather than taxonomically and have a high GC DNA base content. They are taxonomically classified as *Actinobacteria*[4]. *Lactobacillus,* one of the most well-known genera of LAB, has a low GC DNA base content and is taxonomically classified as *Firmicutes*. Both are strictly fermentative (hetero- or homo-fermentative) and many species are known to produce antimicrobial substances, such as hydrogen peroxide (H_2_O_2_), acetic acid, and in some cases, antimicrobial peptides known as bacteriocins [5-7].

*Lactobacillus* and *Bifidobacterium* have relatively small genomes, ranging from 1.2 to 3.4 mega base-pairs (Mbp) and harboring approximately 1100 to 3400 predicted genes [8]. The genome degradation and gene loss in *Lactobacillales* through evolution have been substantial, with 600 to 1200 genes lost since their divergence from the *Bacillus* ancestor, while fewer than 100 genes have been gained [1,2]. Many enzymes are among these lost genes, rendering limited biosynthetic capacities [9]. The genes gained, most of which belong to transport systems and the degradation of carbohydrates, peptides, and amino acids, facilitate nutrient uptake [9]. To our knowledge, no study of these mechanisms has been reported for *Bifidobacterium*, however since they live in similar environments, similar degradation should be expected. The core genes in *Bifidobacterium* encode proteins involved in housekeeping functions such as replication, transcription, and translation, but also in functions related to adaptation to a particular niche such as carbohydrate metabolism, cell envelope biogenesis, and signal transduction [10]. Lactobacilli and bifidobacteria have been investigated for use in food fermentation and preservation; however, in recent years selected species within both genera are being investigated for clinical applications in treating gastro-intestinal and vaginal infections [11]. Interestingly, LAB can be involved in a process known as proto-cooperation, in which two or more of the bacteria work together to produce the enzymes needed for the synthesis of important substances [12,13].

This article focuses on a symbiotic LAB microbiota composed of nine *Lactobacillus* and four *Bifidobacterium* species from the honeybee *Apis mellifera*[14,15]*,* in which the majority are described as novel species (data in publication) and specifically on the extra-cellular proteins they produce. This microbiota were first discovered in the honey crop of *Apis mellifera* as key bacteria in honey production [14], and similar strains were subsequently found in all nine recognized *Apis* species and stingless bees in all continents [15]. It is interesting that these 13 LAB species are always found in the honey crop of honeybees regardless of the bees’ geographical location [15-17], as this indicates that the insect and bacteria have co-evolved throughout history. The LAB microbiota are symbiotic with each other, with their host, and with the visited flowers, defending their niche against bacteria and yeasts introduced by nectar foraging and food intake [15]*.* We recently demonstrated that these bacterial symbionts have antimicrobial action against two severe bee pathogens, *Paenibacillus larvae,* which is known to cause American foulbrood disease, and *Melissococcos plutonius*, the cause of European foulbrood disease [15-18]. These qualities are certainly due to the production of a number of metabolites such as lactic acid, formic acid, di-acetyl, acetic acid, and H_2_O_2_, which could also contribute to their host defense [15,16,18] (and data in publication).

Because of selective pressure from environmental changes in their niche, LAB evolved stress response systems and defenses to allow them to grow and survive in harsh conditions. The honey crop, with its constant nectar flow, high osmotic pressure, and presence of microorganisms introduced by foraging is the ideal environment for these systems to be activated. These systems in these conditions rely on specific gene expressions in different cell processes, such as extra-cellular proteins and peptides, to deal with these harsh environmental conditions [19]. In general, LAB can produce great amounts of cell surface and extra-cellular proteins such as bacteriocins, molecular chaperones, enzymes, lipoproteins, and surface layer proteins [6,20] that are involved in varying cell processes. Surface layer or extracellular proteins are essential for niche protection, and their survival forms part of the proteome known as the “secretome” [21].

From our previous research we have seen that these symbiotic LAB species possess antimicrobial properties against bee pathogens and other microorganisms introduced by nectar foraging and they work together synergistically as a defense system [15,18]**.** In this work we investigate whether this activity could be attributable to any secreted proteins. To that end, we identify extra-cellular proteins from each *Lactobacillus* and *Bifidobacterium* spp. from the honey crop separately under microbial stress in order to understand their ecological roles as antimicrobial barriers against incoming threats and their roles in honey production.

## Results

The honey crop *Lactobacillus* Fhon13N, Biut2N, Hma8N, Bin4N, Hon2N, Hma11N, Hma2N, Bma5N, and *Lacobacillus kunkeei* Fhon2N have genome sizes ranging from 1.5 to 2.2 Mbps, and the number of predicted proteins ranges from 1330 to 2078 (Table 1). The fraction of predicted proteins in these strains with known function is on average 71%, the fraction without known function but similar to other known proteins is on average of 26%, and proteins without known function or similarity are on average 4%. The honey crop *Bifidobacterium* Bin2N, Bin7N, Hma3N, and *Bifidobacterium coryneforme* Bma6N have genome sizes ranging from 1.7 to 2.2 Mbps, and the number of predicted proteins ranges from 1386 to 1836 (Table 1). The fraction of predicted proteins in Bin2N, Bin7N, Hma3N, and *B. coryneforme* Bma6N with known function is on average 69%, without known function but similar to other known proteins is on average 26%, and proteins without known function or similarity are on average 5%. Further genomic data and analysis on these 13 LAB species will be covered in full detail in another paper.

**Table 1:** Genomic characteristics of the 13 LAB symbionts from the honey crop

	**Genome size (Mb)**	**Total ORFs**	**ORFs - with assigned function (%)**	**ORFs - without assigned function, with similarity (%)**	**ORFs - no similarity or assigned function (%)**
***Lactobacillus***					
**Fhon13N**	1.5	1 330	72	25	4
**Fhon2N**	1.6	1 504	73	24	3
**Bin4N**	1.8	1 715	73	22	5
**Hon2N**	1.8	1 707	71	24	5
**Hma8N**	2.1	2 078	68	29	3
**Bma5N**	2.0	1 929	69	27	4
**Hma2N**	2.2	2 066	69	28	3
**Biut2N**	2.1	2 037	70	27	3
**Hma11N**	1.7	1 585	71	25	4
***Bifidobacterium***					
**Bin2N**	2.1	1 740	67	27	6
**Bin7N**	2.1	1 718	69	26	5
**Hma3N**	2.2	1 836	68	26	6
**Bma6N**	1.7	1 386	73	23	4

**Figure 1:**
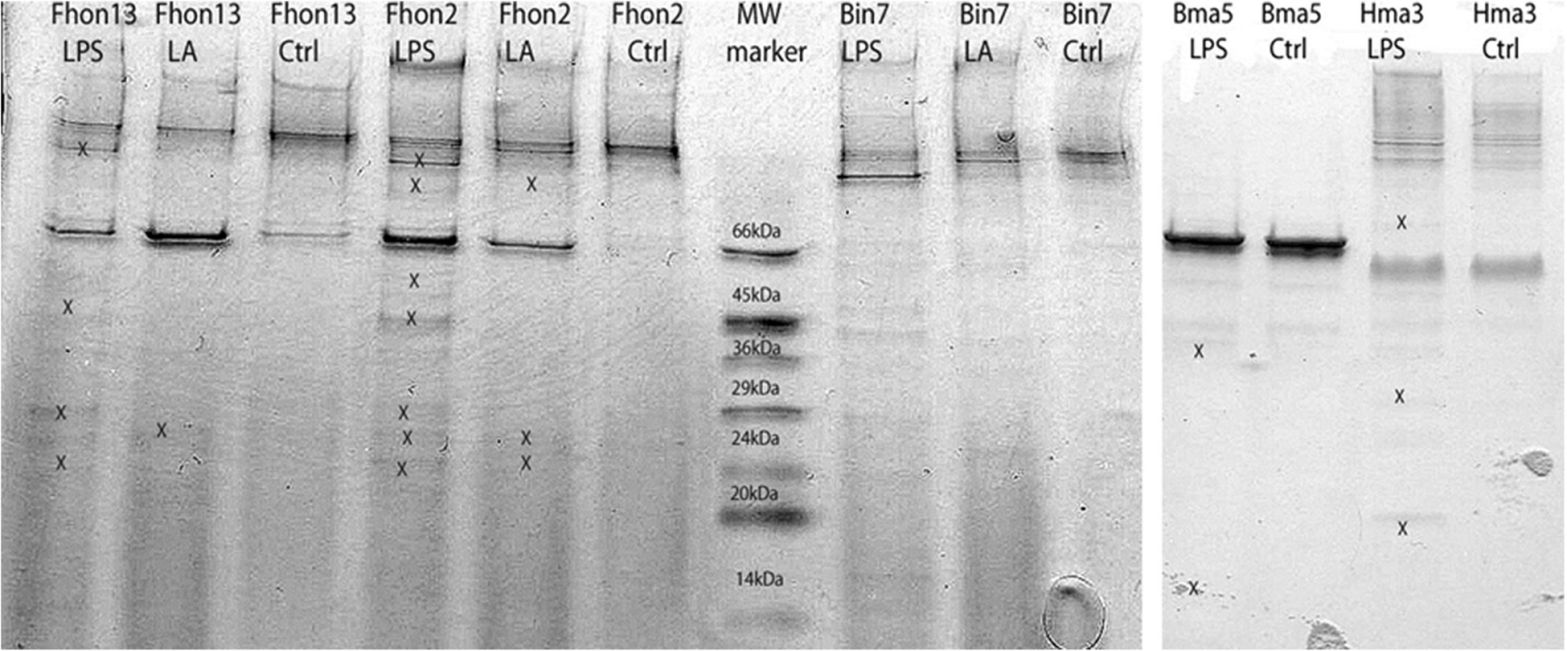
**Tricine-SDS-PAGE analysis of extracellular proteins and peptides from some of the LAB strains during stressed and un-stressed conditions.** Lane 1- *Lactobacillus* Fhon13N stressed with LPS, Lane 2- *Lactobacillus* Fhon13N stressed with LA, Lane 3- *Lactobacillus* Fhon13N unstressed, lane 4- *L. kunkeei* Fhon2N stressed with LPS, lane 5- *L. kunkeei* Fhon2N stressed with LA, lane 6-*L. kunkeei* Fhon2N unstressed, lane 7- molecular weight marker, lane 8- *Bifidobacterium* Bin7N stressed with LPS, lane 9- *Bifidobacterium* Bin7N stressed with LA and lane 10- *Bifidobacterium* Bin7N unstressed. The second gel is as follows: Lane 1- *Lactobacillus* Bma5N stressed with LPS and lane 2- *Lactobacillus* Bma5N, lane 3- *Bifidobacterium* Hma3N stressed with LPS, lane 4- *Bifidobacterium* Hma3N unstressed. Marks of X are an indication of where a band was cut and analyzed with MS.

An overview of the results of extra-cellular peptides and proteins from each LAB during microbial stress is shown in Figure 1 and in Table 2. Each of the 13 species and the extra-cellular proteins they produce are depicted more thoroughly in the Additional file 1: Table S1-S9. Putative identification and function were achieved from searches in NCBI (non-redundant database), InterProScan (default database), and Pfam (default database). We identified a vast range of extra-cellular proteins from 10 of the 13 LAB spp., but the majority of the proteins produced had unknown functions. Most of the identified proteins were enzymes, S-layer proteins, DNA chaperones, bacteriocins, and lysozymes (Table 2).

**Table 2:** An overview of all extra-cellular proteins synthesized during stress conditions (LPS, LA), from all 13 LAB spp

	**Peptides with unknown function**	**Peptides with enzymatic function**	**S-layer proteins**	**Chaperones and stress response proteins**	**Bacteriocins and lysozymes**	**Other**	**Total proteins produced**
*Lactobacillus*							
Fhon13N	4	4	0	0	1	0	9
Fhon2N	17	3	0	0	1	3	24
Bin4N	5	7	0	1	0	9	22
Hon2N	4	26	0	5	1	10	46
Hma8N	0	0	0	0	0	0	0
Bma5N	0	2	1	0	1	0	4
Hma2N	0	8	2	0	0	2	12
Biut2N	3	0	0	0	0	0	3
Hma11N	0	1	2	1	0	0	4
*Bifidobacterium*							
Bin2N	2	5	0	0	0	0	7
Bin7N	0	0	0	0	0	0	0
Hma3N	5	4	0	2	1	0	12
Bma6N	0	0	0	0	0	0	0

Tricine-SDS-PAGE analysis showed that differences between stressed and un-stressed protein production varied greatly between both *Lactobacillus* and *Bifidobacterium* genera, and also between each individual LAB (Figure 1). Figure 1 shows the differences in the extra-cellular protein abundance of stressed lactobacilli *L. kunkeei* Fhon2N and *Lactobacillus* Fhon13N compared to unstressed controls; there were no differences in bands between stressed and un-stressed controls for the *Bifidobacterium* Bin7N. Figure 1 shows small differences between stressed and un-stressed *Lactobacillus* Bma5N and *Bifidobacterium* Hma3N. Many of the obvious protein production differences between stressed and un-stressed controls were from lower molecular-weight peptides, while similar banding patterns were seen in the higher molecular weight section. Some of the similar bands are seen to be lighter or darker indicating that there may be up- or down- regulation of genes.

Mass spectrometry and peptide mass fingerprinting identified differences between each studied LAB in the type and number of proteins produced (Table 2, Additional file 1). We noticed that in some cases, some LAB produced many proteins (*Lactobacillu*s Hon2N, Bin4N, and *L. kunkeei* Fhon2N), while others produced none at all (*Lactobacillus* Hma8N, *Bifidobacterium* Bin7N and *B. coryneforme* Bma6N). We also observed differences between the stressors lipopolysaccharide (LPS), lipotechoic acid (LA), and peptidoglycans (Pgn), and in the duration the LAB were stressed (Additional file 1). LPS was the most effective stressor, while LA was effective in 3 cases (Hon2N, Bma5N, and Bin2N) (Additional file 1). The peptidoglycans stressors were not effective in any of the 13 LAB protein productions. The extra-cellular secretion of enzymes was high in all 10 LAB, while the production of proteins with unknown function was highest with *L. kunkeei* Fhon2N (Table 2 and Additional file 1). About 3% of the predicted genes in *L. kunkeei* Fhon2N were classified as gene products without unknown function or similarity (Table 1). None of the *Bifidobacterium* spp. produced bacteriocins, SLPs, or chaperones except *Bifidobacterium* strain Hma3N, which produced one putative lysozyme/bacteriocin and two chaperones (Table 2, Additional file 1). *Lactobacillus* Biut2N was unique in that it only produced unknown proteins under stress conditions. (Table 2). We also identified that 16% of the known extra-cellular proteins we discovered during stress had an identified signal peptide when checked with InterproScan.

**Figure 2:**
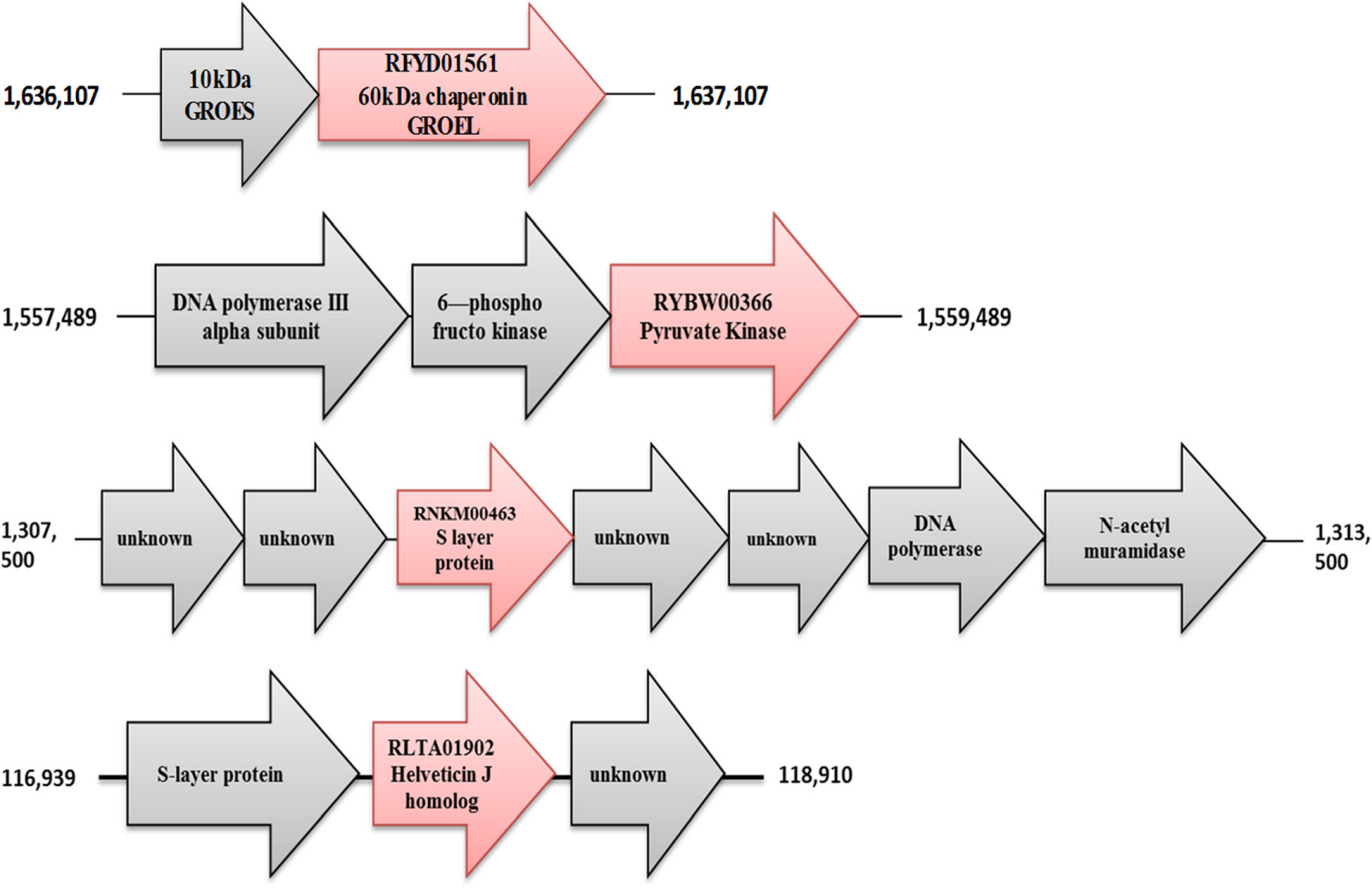
**Putative predicted operons: Predicted operon examples for four of the extra cellular proteins found in the LAB spp.** Each picture displays the surrounding genes or operon as well as gene location. The first example is a 60 kDa chaperonin (RFYD01561, [GenBank: KC776105]) predicted operon from *Lactobacillus* Bin4N, involving the cistrons that form the predicted operon. The red arrow is the extra-cellularly identified chaperonin GroEL, while the grey arrow is the other predicted cistron that forms the putative operon (chaperonin GroES). The red arrow is the extra-cellularly produced enzyme pyruvate kinase while the grey arrows are the other predicted cistrons that form the putative operon. The second is an example of enzyme pyruvate kinase (RYBW00366, [GenBank: KC789985]) predicted from *Lactobacillus* Hon2N operon, involving cistrons that form the predicted operon. The third set of arrows is an example of an S-layer protein (RNKM00463, [GenBank: KC776070]) predicted from a *Lactobacillus* Hma11N operon, involving the genes that form the predicted operon and the surrounding genes of interest. Interestingly this putative SLP is not part of an operon but surrounded by two operons. The predicted operon can be seen in grey. The red arrow displays an example of the SLP that is extra-cellularly produced. The last set of arrows displays the putative surrounding genes for the Helveticin J homolog (RLTA01902, [GenBank: KC776075]) that was identified in *Lactobacillus* Bma5N. This putative bacteriocin (red arrow) does not form part of an operon but is surrounded by an S-layer protein and unknown protein (grey arrows).

Predicted operons of interesting extra-cellular proteins are shown in Figure 2. A predicted putative operon of Hsp60 chaperonin GroEL (RFYD01561; [GenBank: KC776105]) from *Lactobacillus* Bin4N is displayed in Figure 2. Figure 2 also shows the predicted putative operon for the enzyme pyruvate kinase that was identified extra-cellularly from *Lactobacillus* Hon2N (RYBW00366; [GenBank: KC789985]). Examples of single genes that were not found to be part of a putative operon were RLTA01902 (GenBank: KC776075) (helveticin J homologue, Max ID 51%) from Bma5N, N-acetyl muramidase (ROMW00411); (GenBank: KC776084) from *L. kunkeei* Fhon2N and the S-layer protein RNKM00463 (GenBank: KC776070) from Hma11N. This SLP is however surrounded by two operons, which are shown in Figure 2. The helveticin J homologue was expressed when stressed with LPS and LA, did not form part of a putative operon, but was instead flanked by an S-layer protein and a protein with unknown function.

## Discussion

Lactobacilli and bifidobacteria have an essential role in the health of both humans and animals through their interaction with their surrounding environment, and by their production of primary and secondary metabolites including antimicrobial substances [22,23]. The genomes of the 13 honeybee-specific LAB investigated here are typical small genomes characteristic for bacteria within LAB that have been sequenced by now when searched in NCBI BLAST (Table 1). This indicates an adaptation to the nutrient-rich environment in the honey crop and a possible proto-cooperation. A strain that probably progressed far in adaptation and genome degradation is *B. coryneforme* Bma6N. It has an unusually small genome for a *Bifidobacterium* and could have a specialized function in the honeybee microbiota. Furthermore, its protein pattern does not change when incubated with any of the tested microbial stressors (Table 2). Two other LAB, *Lactobacillus* Hma8N and *Bifidobacterium* Bin7N (Figure 1 and Table 2) do not display any changed extra-cellular protein pattern upon co-incubation, and might have other functions in the niche such as production of other metabolites that were not tested in this study. These LAB may just be commensals and not have any other function besides from inhabiting the honey crop and biofilm formation. More experiments will have to be done to confirm this however.

Extra-cellular proteins may play a significant role in the antimicrobial or immunological response against food spoilage microorganisms and pathogens invading the honey crop, but also aid the uptake of nutrients by enzymatic breakdown. It is well known that LAB produce bacteriocins which are ribosomally synthesized antimicrobial peptides [24] that are classified into 3 main classes: I (lantibiotic), II (heat-stable non-modified), and III (heat-labile) [5,25]. The fraction of predicted secreted proteins classified as bacteriocins average around 2% in other published *Lactobacillus* genomes but can be as high as 22% in a strain of *Oenococcus oeni*[21]. One of the identified proteins produced by *Lactobacillus* Bma5N (Gene No. RLTA01902 in Additional file 1: Table S5, [GenBank: KC776075]) when stressed with LPS and LA, showed homology (Max ID of 51%) to a known bacteriocin named Helveticin J when compared with other species in NCBI BLAST (Additional file 1). Helveticin J is a Class III bacteriocin that is quite large in size (> 30 kDa) [26] and was described as a heat-sensitive bacteriocin that could inhibit the growth of other *Lactobacillus* species [27]. However, the homologue we found contained no conserved signal peptides when searched through InterProScan, indicating a putative novel bacteriocin. Remarkably, *Lactobacillus* Bma5N was previously shown by us to be one of the most active LAB against the bee pathogen *P. larvae*[18]*.* These earlier observations might have been caused by this putative novel bacteriocin. Most bacteriocins are encoded on plasmids, yet Helveticin J is found chromosomally, and in the case of our helveticin homologue, on the secondary chromosome, not forming part of an operon. Instead the gene is singly located, surrounded by an S-layer protein and a protein with unknown function (Figure 2). There were secreted proteins detected in 7 of the LAB spp. that had no known function (Table 2). Their genes were located in close proximity to peptide efflux ABC transporter ORFs in the genomes, indicating putative novel bacteriocins or antimicrobial proteins. Bacteriocins and ABC-transporter coding genes are commonly seen in close proximity to each other in the same operon [28]. However, we need more research in order to understand their actual function.

The majority of extracellular proteins produced by each honeybee-specific LAB under stress were enzymes (Table 2). However, the enzymes produced are not the same from each strain. An enzyme produced in *Lactobacillus* Fhon13N, Hon2N, and *L. kunkeei* Fhon2N, and *Bifidobacterium* Hma3N when under LPS stress for 1 and 3 days, was N-acetyl muramidase, a hydrolase that acts as a lysozyme (Additional file 1). These extra-cellularly produced lysozymes had conserved signal peptide sequences suggesting there importance as extracellular proteins. Glycoside hydrolases such as muramidases, lactases, and hyaluronidases are a group of enzymes that can be involved in antibacterial activity, and some of these hydrolases have been assigned to Class III of bacteriocins. Muramidases or, lysozymes, can be involved in both gram-positive and gram-negative bacterial cell wall peptidoglycan degradation [29,30]. This suggests a putative function as a bacteriolysin or class III bacteriocin. Interestingly, it has been shown that these muramidases may also interact with the human immune system, acting as immune-adjuvants [6]. It is feasible to assign similar functions for these enzymes in their natural niche, the honey crop in which they may interact with their host (the honeybees), or by enzymatic defense against unwanted introduced bacteria. Again, more research is needed in order to outline their true function.

We noticed that enzymes known to be intra-cellular, such as glucose 6-phosphate dehydrogenase (GAPDH) and lactate dehydrogenase (LDH) appeared in extra-cellular supernatants of *Lactobacillus* Fhon13N, Bin4N, Hon2N, Bma5N, Hma2N, *L. kunkeei* Fhon2N, and *Bifidobacterium* Bin2N (Additional file 1). One possible explanation for these results is cell lysis causing intracellular proteins to leak. LDH and GAPDH are two important enzymes involved in carbohydrate metabolism, most noticeably in the process of glycolysis and lactic acid production in LAB. Research has shown that glycolytic and ribosomal proteins are found on the bacterial cell-surface and are also internally expressed, however it is still unknown how or why these proteins are expressed and reach the cell surface. It is hypothesized that these proteins, once they are localized on the surface, could develop different functions other than those known and might become “moonlighters” [31,32]. For example, Kinoshita and colleagues discovered GAPDH expressed on the surface of *Lactobacillus plantarum* was involved in the adhesion of the bacteria to colonic mucin [33]. This could be the case for some of the secreted proteins we found that are known to be intra-cellular (Additional file 1). We have previously shown that the LAB symbionts inhabit their niche in biofilms [15], however it is still unclear what substances are involved in their formation. We hypothesize that these enzymes may be extra-cellularly secreted and are likely involved in synthesizing the building blocks of biofilm formation.

**Figure 3:**
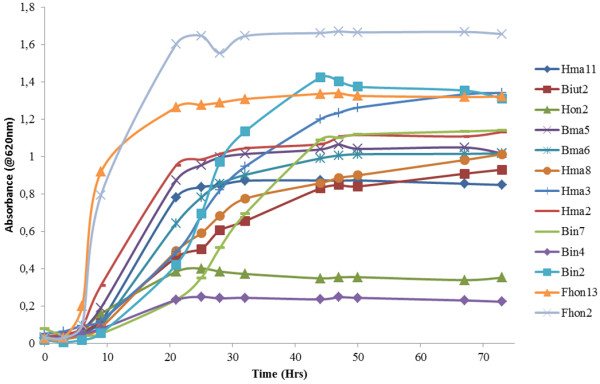
**Growth kinetic analyisis of all 13 species of LAB 0–3 days.** LAB were grown on MRS agar and changed into new MRS medium and kinetic growth curves were measured in triplicate. All 13 LAB were measured from 0 to 72 hours at 620nanometers. This was performed to discover the different growth phases of the LAB and when each enters early stationary phase.

We also saw in some cases extra-cellular LSU and SSU ribosomal subunits were produced (Additional file 1). This could also be due to the bacterial cell lysis however since these LAB are not entering the death phase during this time it is probably not likely (Figure 3). Some leakage could possibly be occurring however. Two of the LAB (Bin4N and Hon2N) produced more extra-cellular ribosomal subunits and both are slow growing compared to the other LAB symbionts. This could suggest some lysis was occurring however it is normal for these LAB species to grow slowly as they are closely related species [15] (Figure 3, Additional file 1). Another possible explanation is that maybe this extra-cellular DNA is used in the formation of the biofilm that these LAB use to interact and survive.

S-Layer proteins (SLP) are one of the most common membrane surface structures in bacteria and make up a large percentage of the total protein content of the bacterial cell, indicating that they are important in structure and/or function [34,35]. Nevertheless, the functions of SLPs have been described only hypothetically. Åvall-Jääskeläinen and Palva (2005) argued that SLPs were involved in protective cell coats, trapping molecules and ions, and acting as structures for adhesion and cell surface recognition [36]. We detected secretion of SLPs only from some lactobacilli (Hma2N, Hma11N, and Bma5N) (Table 2). Each identified SLP contained a conserved SLAP domain determining its surface-layer identification. However, the SLPs that were produced did not form part of a putative operon, but instead were found as single genes in between two other putative operons in the genomes. The putative operons surrounding the SLP can be seen to follow a specific gene organization, with a gene coding for N-acetyl muramidase and an unidentified cytosolic protein (Figure 2). We suggest that the SLP in this case may act as a protective layer to inhibit the muramidases destroying the cell wall of the strain that produced it. Poppinga and colleagues identified an SLP in *P.larvae,* which causes American foulbrood disease in *A. mellifera*. They suggested that the pathogens secrete this SLP to aid adherence of the parasite to the bee gut [37]. It has been shown that specific LAB strains can compete for the same receptors in humans as other pathogens in the gastrointestinal tract by competitive exclusion [38,39]*.* We know that the LAB symbionts anchor themselves to the crop with structures resembling a mixture of proteins and exo-polysaccharides [15], therefore SLPs may be involved in biofilm formation and take part in the adhesion of the bacteria to the honey crop wall. No S-layer proteins have been annotated in any of the draft *Bifidobacterium* genomes. Possible reasons for the lack of SLPs in the bifidobacteria might be that they use other mechanisms such as sugars or other lipoproteins for adhesion and protection purposes [40]. The fact that not all of the honeybee LAB symbionts produce these proteins indicates that they are most likely working together in symbiosis to protect themselves in their environment.

Molecular chaperones (stress proteins) were produced from a number of the LAB symbionts (Table 2). LAB, like other bacteria have developed systems to sense stress situations from sudden environmental changes and other invading organisms [41]. Chaperones are transcriptional regulators and can co-ordinate the expression of the genes involved in a stress response and improve LAB stress tolerance [19]. Molecular chaperones also have a number of other functions, for example protein folding, preventing protein aggregation, targeting proteins for secretion, and the transfer of peptides across membranes [41,42]. Hsp60 (GroEL) and Hsp70 (DnaK) are both well-conserved proteins in lactobacilli and bifidobacteria and are most efficiently induced by heat [43,44]. Some of the LAB symbionts produced DNA chaperones extra-cellularly (*Lactobacillus* Hon2N, Hma11N, Bin4N, and *Bifidobacterium* Hma3N, which produced DnaK or GroEL, Additional file 1). *Bifidobacterium* Hma3N produced both when stressed with LPS for 3 days, while *Lactobacillus* Hon2N produced both of the chaperonins DnaK and CsaA, and also the two universal stress proteins UspA when stressed with LPS for 1 day (Additional file 1). These molecular chaperones are usually seen within the bacterial cytosol, however there have been reports showing that bacteria can produce them extra-cellularly as “moonlighting” proteins [45]*.* The LAB may produce enzymes extra-cellularly to interact with their host, since many adhesion molecules are needed in such a harsh environment. Bergonzelli *et al.* reported that chaperonin GroEL of *Lactobacillus johnsonii* has been found on the surface of the cells and could interact with *Helicobacter pylori*, indicating a competition for binding sites in humans [41]. However, the LAB symbionts may release the chaperonins to aid in the folding of other secreted proteins that are more typically their function [40]. We did notice that 16% of the known proteins discussed in Table 2 had signal peptide sequences however many more of the proteins produced can be transported from the cell without the need for these signals, for example bacteriocins, DNA chaperones and some enzymes. More research should be performed to investigate the mode of extra-cellular transport in order to understand the functions of these produced proteins.

We can see from the majority of the extra-cellularly produced proteins secreted by the 13 symbiotic LAB, were produced under stress by LPS, which was extracted from *Pseudomonas aeruginosa*. Interestingly, species within the genus *Pseudomonas* are often isolated from flowers and introduced into bees and their crop by nectar foraging [15]. Our results show that lipotechoic acid (LA) was not as an effective stressor as LPS, however it is important to remember that during stress many LAB produce different proteins, but the production of these proteins can differ depending on the stress [19]. This is outlined in our results and is important to remember when performing any other future experiments.

## Conclusion

Strains within LAB are commonly applied for their beneficial health effects and in the defense against pathogens as many are known to have immune-stimulatory functions [22,36]. Due to the increased number of antibiotic-resistant pathogens in infection, novel strategies must be found to combat this problem. Since ancient times, honey has been used as a folk medicine due to its antimicrobial activity and has been used for wound management due to its biochemical and antimicrobial properties [46,47]. The LAB used in the present study are honeybee symbionts co-existing within the honey crop in huge numbers and involved in honey production. It is feasible to believe that their secreted substances lead honey’s antimicrobial activity. Therefore LAB could play an essential role as a future alternative tool against infections. It is clear from the results that the symbiotic *Lactobacillus* and *Bifidobacterium* species in the honey crop of *A. mellifera* play a vital role in defending their niche and honey production. Differences in protein production could indicate that these bacteria are involved in proto-cooperation and need each other to survive in the honey crop. Further research must be performed to identify the antimicrobial effects of these known and unknown extra-cellular proteins and how they can be applied against infections.

## Methods

### Bacterial strains and culture conditions

*Lactobacillus* Fhon13N, Hma8N, Bin4N, Hon2N, Hma11N, Hma2N, Bma5N, and Biut2N, *L. kunkeei* Fhon2N, and *Bifidobacterium* Bin2N, Bin7N, Hma3N, and *B. coryneforme* Bma6N*,* used in this study were isolated from the honey crop of the western honeybee subspecies *Apis mellifera mellifera*. All collected bees originated from the same apiary in an *A. m. m* protected area in Hammerdal, Jämtland, in northern Sweden where they were part of a conservation project called NordBi ( http://www.nordbi.org/). Bacterial strains were isolated at different occasions during the summer season as we know that concentrations of single members of LAB microbiota vary depending on nectar foraging and other identified factors. The identity of bacterial isolates was established by sequencing the 16S rDNA genes of 370 isolates as previously described [14,15]. All 13 LAB were grown in MRS (DeMan, Rogosa & Sharpe, Oxoid, UK) broth, supplemented with 2% fructose, 0.1% L-cysteine, and incubated until early stationary phase at 35°C (See Figure 3). There was some variation between all 13 LAB strains incubation time as some entered early stationary phase later than others (Figure 3). They were re-incubated to early stationary phase 3 times so LAB could adjust to MRS medium. Microbial stress experiments could then be performed,

### Microbial stress

Each bacterium was re-suspended in filtered (10 K Amicon ultra 0.5 ml centrifugal filters, Millipore, Ireland) MRS medium. Microbial stressors, Peptidoglycan from *Saccharomyces cervisiae* and *Micrococcus luteus* (2 mg/ml, Sigma-aldrich, USA), Lipotechoic Acid from *Streptococcus pyogenes* (2 mg/ml, Sigma-aldrich, USA), and Lipopolysaccharide from *Pseudomonas aeruginosa* (2 mg/ml, Sigma-aldrich, USA) were added. Bacteria were stressed by co-incubation with the mentioned microbial stressors from 0 to 1.5 or 3 days at 35°C.

Samples were centrifuged at 13 000 rpm for 10 minutes. Supernatant was taken from each tube and added to 30 K Amicon ultra centrifugal filters (Millipore, Ireland) and centrifuged for 10 minutes at 13 000 rpm. 0.2 M Tris–HCl (pH 8.3) was added to the filter and samples were centrifuged as before. This step was repeated once and 6 M urea (in 0.2 M Tris–HCl) was added to the filter and centrifuged as before [48,49]. Samples were frozen at −20°C until further use. Unstressed bacteria (without LPS or LA) were also concentrated in accordance with the same procedure to be used as controls.

### Tris-tricine SDS-PAGE and mass spectrometry

To separate proteins from the stressed and unstressed bacteria, Mini-PROTEAN 10% to 20% Tris-Tricine precast gels (BioRad, USA) were used as per original protocol [50]. Concentrated samples were run at 105 V as previously described. Gels were stained with Biosafe Coomassie (BioRad, USA) following the manufacturers’ instructions. Controls and stressed samples were run together and compared. Differences between band patterns originating in the same bacterium were compared and bands seen only in stressed bacterial samples were cut and further analyzed. A molecular weight MW marker was used (Bio-Rad, USA): 14–66 kDa. Gel bands were prepared for mass spectrometry as outlined in the paper by Shevchenko *et al.* 1996*,* with some modifications. Gel bands were first de-stained and shrunk by the continuous addition of 50 to 100 mM Ambic (NH_4_HCO_3_) (Sigma-Aldrich, USA) and 50% Acetonitrile (Sigma-aldrich, USA) until all Coomassie had been removed from the gel pieces. Gel pieces were then prepared as per protocol [51]. The tryptic peptides from the secreted proteins were run on an Agilent HPLC on a C18 reverse phase column (75 μm × 150 mm, particle size 3 μm). Total run time was 90 min and flow rate 300 nl/min. Buffers used for gradient were 0.1% formic acid in water (buffer A) and 0.1% formic acid in acetonitrile (buffer B). The buffer mixing was 5 min 5% buffer B, followed by 5% to 45% buffer B in a linear gradient for 50 min, followed by 45% to 80% buffer B in a linear gradient for 5 min. The 80% of buffer B was then kept for 15 min and then rapidly back to 5% buffer B for the final 15 min. The fractions from HPLC were loaded on an LCQ Deca XP Plus Ion trap mass spectrometer (ThermoScientific).

### Genomic sequencing, bioinformatics, and peptide mass fingerprinting

Genomic DNA were prepared from all 13 LAB depicted earlier and sequenced at MWG Eurofins Operon (Ebensburg, Germany) using Roche GS FLX Titanium technology from Roche (Basel, Switzerland). For each genome, a shotgun library was constructed with up to 700 000 reads per segment and was generated by sequencing in 2 × ½ segment of a full FLX + run. Each genome had an 8 kpb long-paired end-library constructed. Approximately 300 000 true paired end-reads, sequence tags, and scaffolds with GS FLX + chemistry using 2 × ½ segment of a full run were generated. Clonal amplification was performed by emPCR in both library types. The sequencing was continued until 15- to 20-fold coverage was reached.

The obtained reads were assembled by the software Newbler 2.6 from Roche (Basel, Switzerland). ORF prediction and automated annotation was performed at Integrated Genomics Assets Inc. (Mount Prospect, Illinois, USA). In ORF prediction three different software packages were used: GLIMMER, Critica, and Prokpeg. Automated annotation was performed with the ERGO algorithms (Integrated Genomics Assets Inc. Mount Prospect, Illinois, USA).

The resulting mass spectra-files obtained from the mass spectrometry analysis were searched using MASCOT against a local database containing the predicted proteome of the 13 LAB [52]. We used a cut-off Ions score of 38 as a value for determining that the protein was identified. Individual ion scores greater than 38 indicated identity or extensive homology (*P* < 0.05) of the protein. Protein sequence similarity searches were performed with software BLASTP in the software package BLAST 2.27+ against a non-redundant protein database at NCBI [53,54], Pfam (default database) [55], and InterProScan (default databases) [56,57]. Expressed proteins identified by peptide mass fingerprinting were manually re-annotated.

### Identification of predicted operons

Operon prediction was achieved with the MolGen Operon Prediction Tool [58]. The sequenced and annotated genomes, in Genbank file format, were run separately with default settings. The *rho*-dependent transcription terminators were predicted by using the TransTerm software [58].

### Availability of supporting data

The 16S gene sequences for all 13 LAB strains can be found in one of our earlier papers [15]. The datasets supporting the results in this article are available with ProteomeXchange Consortium ( http://proteomecentral.proteomexchange.org) via the PRIDE partner repository [59] with the dataset identifier PXD000187 and DOI PXD000187/PXD000187 with PRIDE accession numbers 28788–28855.

The accession numbers of the identified proteins can be found within this article and its supplementary information (See Additional file 1: Tables S1-S9) and are available through NCBI GenBank database [60].

## Competing interests

A. Vasquez and T. Olofsson are the founders of, and hold stock in, ConCellae AB, a spin-off university-based company that develops and markets functional food and medical products. A. Vasquez and T. Olofsson are inventors of three patent applications related to the commercial uses of the 13 LAB. Neither the present study nor any other research activity at Lund University has been funded by ConCellae AB. Therefore, the authors declare no competing interests concerning this work.

## Authors’ contributions

EB was responsible for performing experiments, interpreting MASCOT and genomic data, identifying proteins, designing figures, and writing the majority of the manuscript. MA was involved with genome analysis, protein annotation, putative operon prediction, MASCOT interpretation, figure design, and writing of manuscript. TCO was involved in the design of project, the collection of honeybee colonies from North Sweden, the isolation of LAB spp. from honeybees, and the initiation of the LAB genome sequencing, and also contributed to the writing of the manuscript. CK and JM were involved in designing the project, MASCOT data interpretation, and Mass spectrometry. AV initiated the project, designed the experimental trials, and developed the methods used in the study in collaboration with TO and JM. She supervised the project and took part in writing the manuscript. All authors read and approved the final manuscript.

## Supplementary Material

Additional file 1: Tables S1-S9 This file contains 9 excel spreadsheets (XLSX-format) of each LAB that produced extracellular proteins. Each spreadsheet is labeled by the bacteria it represents e.g. *Lactobacillus* Fhon13N, Bin4N, Hon2N, Bma5N, Hma2N, Hma11N, *L. kunkeei* Fhon2N and *Bifidobacterium* Bin2N, and Hma3N. Each table contains the stressor, gene number & size, GenBank Accession Number, MASCOT ion score with sequence coverage and No. of peptide matches, putative function and finally closest identified organism, accession number, Query alignment, Max identity, E-value and possession of a signal peptide of each predicted protein from NCBI non-redundant database. Click here for file
